# Parental socialization of guilt and shame in early childhood

**DOI:** 10.1038/s41598-023-38502-1

**Published:** 2023-07-20

**Authors:** Milica Nikolić, Eddie Brummelman, Bram Orobio de Castro, Terrence D. Jorgensen, Cristina Colonnesi

**Affiliations:** Research Institute of Child Development and Education, University of Amsterdam, Amsterdam, The Netherlands

**Keywords:** Human behaviour, Psychology

## Abstract

Self-conscious emotions emerge early in human development and they help children navigate social relationships. Little is known about the socialization of self-conscious emotions in early childhood. We theorized that parental mental state language use and warmth would be important for young children’s self-conscious emotions and their consequent prosocial behaviors. Ninety-eight children residing in the Netherlands (52% girls) aged 2–5 (*M* = 48.66 months, *SD* = 13.50 months) visited the research lab with one parent. First, we observed parental mental state language and warmth. Afterward, children were led to believe that they caused a mishap (i.e., accidentally breaking the experimenter’s favorite toy) to evoke their guilt and shame, which we micro-coded. In subsequent tasks, we observed children’s helping behaviors toward the experimenter. We found that the combination of frequent parental mental state language and high warmth was associated with children’s quicker helping to the previously harmed experimenter across toddlerhood and early childhood. More guilt was related to more helping whereas more shame-like avoidance was related to less helping. Our findings based on the sample of Dutch parents and children suggest that, parental frequent mental state talk, in combination with high warmth, may promote children’s ability to repair social relationships and behave prosocially after mishaps.

## Introduction

Everyone makes mistakes—we accidentally break someone’s valuable possession or hurt a person we care about. In these situations, our relationships with other people are at stake. Self-conscious emotions, such as guilt and shame, motivate behaviors that help or hinder reconciliation after such transgressions. These emotions emerge early in human development and they help children navigate their social relationships from an early age^1,2^. Surprisingly, little is known about the socialization of self-conscious emotions in early childhood although it is widely accepted that the roots of human social behaviors lay in the early parental socialization of emotions^3,4^. The current study investigated whether parental warmth and mental state language are linked to young children’s self-conscious emotions after a mishap and their consequent prosocial acts in a sample of 2–5 years old children from the Netherlands. We focused on the critical developmental phase of early childhood, and we used validated paradigms to evoke and (micro-)code parental behaviors, children’s self-conscious emotions, and children’s prosocial behaviors.

### Self-conscious emotions of guilt and shame

Self-conscious emotions such as guilt and shame typically occur in situations involving a mishap or a transgression of social norms and rules, such as breaking someone’s valuable possession or performing poorly in front of others^5^. In these situations, self-conscious emotions are evoked when a person realizes that there might be a possibility of making a negative impression on other people^6^. Traditionally, self-conscious emotions, such as shame and guilt, have been thought to develop only around the age of three or four, when children can evaluate their actions against the social norms and rules and when they are able to understand they may be evaluated by others^2^. However, recent empirical findings suggest that self-conscious emotions appear earlier than previously thought^7,8^ and that shame and guilt can be experienced and displayed by the age of two^1,9–13^.

Although shame and guilt are elicited by the same kind of situations, they have different consequences. Following a mishap or a transgression, guilt is assumed to motivate the actor to repair their actions^14,15^. A person who experiences guilt feels that their actions were wrong^16^. Because actions can be fixed, guilt results in attempts to resolve the wrong actions by repairing, confessing, explaining the mistake, or apologizing. These behaviors have been observed after a mishap or transgression in toddlers and young children, suggesting that young children may be able to experience guilt^1,9^. A person who experiences shame, however, feels that their whole self is bad and small^14,15,17^. These negative cognitions about the self result in withdrawing or hiding from others, in an attempt to protect the self from additional feelings of failure, as the whole self cannot be easily repaired. These withdrawal behaviors after a mishap or transgression have been observed in toddlers, suggesting that children may be able to experience shame from an early age^1,9^. However, some scholars have argued that these withdrawal behaviors do not reflect shame but fearful avoidance^17^. We therefore refer to these behaviors as shame-like avoidance. Importantly, according to this functionalist view on self-conscious emotions, feeling guilty is supposed to facilitate reconciliation^15,18^, whereas feeling ashamed is supposed to inhibit reconciliation^16,18^. There is empirical evidence that, indeed, guilt is related to increased prosocial behaviors whereas shame is related to decreased prosocial behavior^19^, even in children as young as 2 years old^1,11^.

### Parental influences on children’s self-conscious emotions and prosocial behaviors

Although self-conscious emotions are essential for children’s regulation of social relationships, surprisingly little is known about how these emotions are socialized. Children’s self-conscious emotions are thought to develop through interaction with important others^20^. In early childhood, thus, parents are assumed to play a crucial role in socializing children’s self-conscious emotions^3,4^. Parental socialization of emotions may occur in situations in which children experience certain emotions through parental reactions to children’s behaviors or parental discussion of emotions with children^3^ as well as through parental daily behaviors toward the child in various situations^3,20^.

For the socialization of self-conscious emotions, parental mental state language use and warmth may be particularly relevant. Parental mental state language—using mental state terms (i.e., words referring to emotions, intentions, and cognitions) while talking to the child^21,22^—supports children’s internalization of social norms and rules^23,24^ and helps children learn about themselves and other people^25,26^. This internalization of social norms and rules, as well as thinking about the self and others, is crucial for the experience of self-conscious emotions, including guilt and shame^2^. In addition, parental warmth—showing affection, support, and encouragement to the child—supports children’s developing sense of self-worth^27–29^. Parents who show affection toward their child communicate to the child that they are worthy of attention and love^30^ and the child internalizes the parents’ view of them as worthy^31^, at least in individualistic Western countries^32^. Seeing oneself as worthy or unworthy is a crucial aspect of self-conscious emotions, separating guilt from shame—seeing oneself as worthy is related to guilt, whereas seeing oneself as bad or unworthy is related to shame^14–16^.

In line with these ideas, scholars have suggested that the combination of high parental mental state language and parental warmth may enable children to experience guilt in response to mishaps and transgressions^23,33,34^. This is because talking about own and others’ mental states may increase children’s social understanding and taking others’ perspective^35–37^. When this is done by an affectionate parent who makes the child feels safe and worthy, the child may understand that they hurt another person without feeling bad or unworthy. This understanding may allow the child to be concerned for another person instead of focusing on the negative feelings toward the self^34^. Consistent with this idea, one study found that mothers’ talk about feelings with their child, especially in the context of a supportive mother–child relationship (shared positive affect and secure attachment), is positively associated with mothers’ reports of children’s experiences of guilt after a mishap or transgression^38^.

Less is known about the role of parental mental state language in shame. Although empirical research is missing, the theory on shame suggests that the experience of this self-conscious emotion depends on the reflection upon the self^14,15^. Therefore, it may be expected that parental frequent mentalizing, which enables learning about the self^25,26^, may be related to children’s higher shame. More research has been done on the effects of parental warmth on children’s shame. Because the lack of parental warmth and affection toward the child might make the child feel unaccepted, ignored, or inadequate, children of parents who show a lack of warmth may see their whole selves as unlovable and unworthy and be more prone to experiencing shame after a mishap or a transgression^34^. Indeed, empirical evidence suggests that rejective and negative parents who show little warmth and affection have children who are more prone to experiencing shame^34^.

Parental practices are also important for socializing children’s prosocial behaviors given that children’s prosocial acts develop through social interactions with caregivers^39^. Several studies have investigated the role of parental behaviors in children’s early appearing prosocial acts. For example, parental use of mental state language was positively associated with 18-to-30 month-old toddlers’ helping behaviors^40–42^. Also, maternal sensitivity—that is, warm, contingent, and supportive parenting—has been linked to helping behaviors in 18-month-old toddlers^42^ and preschoolers^43^. Finally, higher mental state language use was related to more children’s prosocial behaviors but only when mothers’ sensitivity was low^42^, suggesting that the combination of different parenting behaviors might matter for children’s prosociality as well.

Notwithstanding the relevance of the above studies, they primarily concern older children and parental reports of own behaviors and children’s self-conscious emotions and not the elicitation of actual self-conscious emotions and parenting behaviors during real social interactions. It is, thus, crucial to examine the associations between parental behaviors and children’s self-conscious emotions in young children using ecologically valid paradigms that allow for the elicitation and systematic observation of parenting behaviors and children’s self-conscious emotions. Furthermore, as theoretical work on self-conscious emotions has suggested, it is important to study the combination of different parental behaviors to disentangle those that are crucial for guilt and those that are crucial for shame.

### This study

To better understand the socialization of young children’s self-conscious emotions, we investigated whether parental mental state language and warmth were associated with children’s guilt and shame-like avoidance in response to a mishap (i.e., breaking the experimenter’s favorite toy) and whether these emotions and parenting behaviors were associated with children’s prosocial behaviors toward the previously harmed experimenter. Additionally, we explored whether these associations were consistent across toddlerhood and childhood (2–5 years old) or whether they were more specific to either toddlerhood or childhood.

We recruited 2–5 years old children and their parents residing in the Netherlands to visit our lab. We elicited parental mental state language and warmth, children’s guilt and shame-like avoidance displays, and children’s helping behaviors in the lab tasks using validated paradigms and we (micro)coded them through observations, which allowed us to investigate real parental and children’s emotional reactions and behaviors. To elicit parental mental state language, we asked parents to read a book to the child and we coded the frequency of parental use of words related to emotions, cognitions, intentions, and wishes. To elicit warmth, we asked parents to help children solve puzzles which were rigged so children experience failure and success, and we coded parental affectionate reactions, support, and encouragement. To elicit children’s self-conscious emotions, we led children to believe that they broke the experimenter’s favorite toy and we micro-coded children’s nonverbal and verbal emotional displays and related behaviors. Finally, to evoke prosociality, we led children to believe that the (previously hurt) experimenter needs help and we coded how quickly the child helps the experimenter.

We hypothesized that frequent parental use of mental state language in combination with high parental warmth would be associated with children’s higher guilt after a mishap and with children’s increased helping. We also hypothesized that more frequent parental mental state language use and low parental warmth would be related to more shame-like avoidance after a mishap and with children’s decreased helping. In addition, we hypothesized that higher guilt would be related to increased helping and higher shame-like avoidance would be related to decreased helping. Finally, we explored whether the expected associations were consistent across toddlerhood (2–3 years old) and childhood (4–5 years old).

## Results

### Preliminary analyses

Means, standard deviations, and zero-order correlations between study variables for the whole sample are reported in Table S4 and for each age group separately in Table S5. Because there were no significant differences between boys and the girls in any of the study variables (all *p*s > 0.211), sex of the child was not included in the analyses.

### Main analyses

#### Testing the hypothesized model for the whole sample

**Figure 1:**
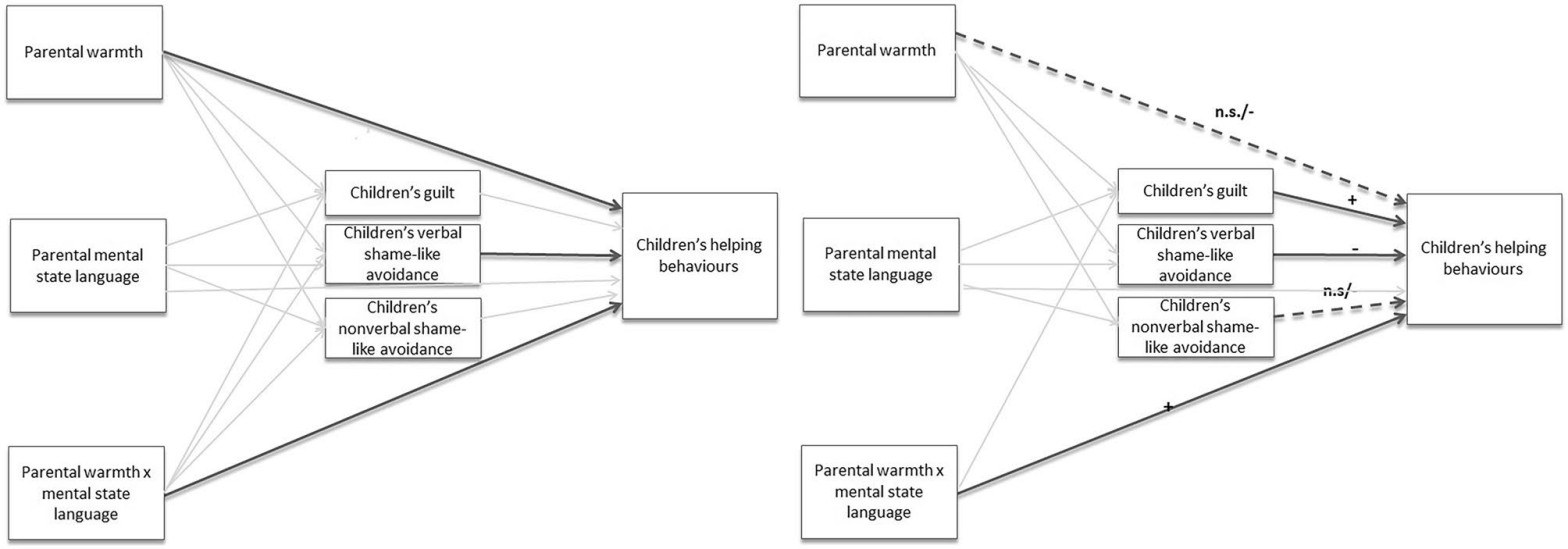
(**a**) Hypothesized model for the whole sample controlling for age. Bold lines represent significant effects. (**b**) Hypothesized model for two age groups. Bold lines represent effects that are significant for both age groups. Dashed lines represent effects that differ for two age groups (the sign of the left shows the direction of the effect for toddlers (2–3 years old) and the sign on the right shows the direction of the effect for older children (4–5 years old).

First, we fitted our hypothesized model in which we controlled for age to test whether the expected associations are found after accounting for the effects of age. This hypothesized model did not fit significantly worse than the saturated model, χ^2^(2) = 0.96, *p* = 0.62; we, therefore, kept this more parsimonious model. CFI = 1.00 and RMSEA = 0.00 [0.00, 0.24] also indicated a close fit. The model’s unstandardized estimates with standard errors and confidence intervals are reported in Table 1 and the significant paths are shown in Fig. 1a.

**Table 1:** Unstandardized point and interval estimates, H_0_ tests, and standardized slopes for the hypothesized model for the whole sample.

	*b*	*SE*	*p*	95% CI	b*
***Direct effects***					
Warmth on guilt	0.24	0.14	.095	− 0.04, 0.52	0.21
Warmth on verbal shame-like avoidance	− 0.19	0.18	.288	− 0.55, 0.16	− 0.13
Warmth on nonverbal shame-like avoidance	0.04	0.04	.318	− 0.03, 0.10	0.10
Warmth on helping	** − 0.71**	**0.29**	**.013**	** − 1.26, 0.15**	** − 0.25**
MSL on guilt	0.01	0.01	.607	− 0.02, 0.03	0.07
MSL on verbal shame-like avoidance	0.01	0.03	.791	− 0.04, 0.06	0.05
MSL on nonverbal shame-like avoidance	0.01	0.01	.223	− 0.004, 0.02	0.19
MSL on helping	− 0.00	0.03	.962	− 0.07, 0.06	− 0.006
Guilt on helping	0.23	0.26	.390	− 0.29, 0.74	0.09
Verbal shame-like avoidance on helping	** − 0.64**	**0.22**	**.003**	** − 1.07, − 0.22**	** − 0.34**
Nonverbal shame-like avoidance on helping	− 0.95	1.01	.345	− 2.92, 1.02	− 0.12
Age on guilt	**0.02**	**0.01**	** < .001**	**0.01, 0.04**	**0.46**
Age on verbal shame-like avoidance	0.01	0.01	.510	− 0.01, 0.02	0.08
Age on nonverbal shame-like avoidance	− 0.001	0.00	.487	− 0.01, 0.002	− 0.09
Age on helping	0.02	0.02	.273	− 0.01, 0.05	0.13
***Moderation effects***					
Warmth × MSL on helping	**0.12**	**0.05**	**.013**	**0.03, 0.22**	**0.30**
MSL on helping at low levels of warmth	** − 0.12**	**0.06**	**.036**	** − 0.24, − 0.01**	** − 0.50**
MSL on helping at high levels of warmth	**0.12**	**0.06**	**.043**	**0.004, 0.24**	**0.50**
Warmth × MSL on guilt	0.03	0.03	.371	− 0.04, 0.09	0.18

*MSL* mental state language, *b* unstandardized slope, *SE* standard error of the unstandardized slope, *p* two-tailed *p* value of the Wald *z* statistic (*z* = *b*/*SE*), *CI* confidence interval around the unstandardized slope, b* standardized slope.

Significant values are in [bold].

**Figure 2:**
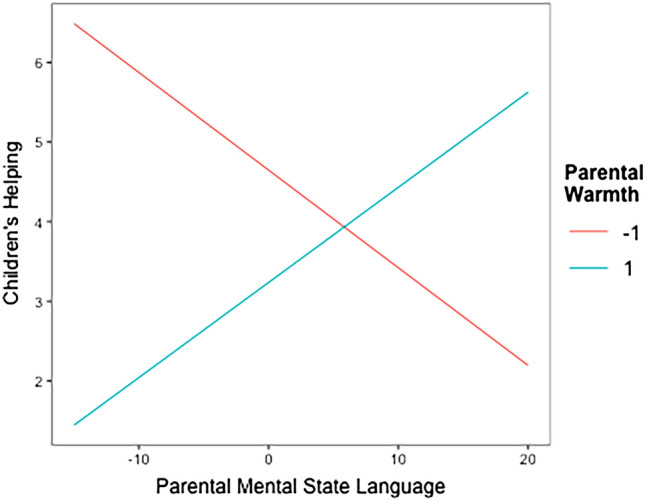
The association between parental mental state language and children’s helping for different levels of parental warmth. Both predictors were mean centered. High and low warmth were visualized using 1 unit above/below the mean. Parental mental state language values are displayed as proportions.

We hypothesized that the combination of frequent parental mental state language use and high warmth would be related to children’s guilt and increased helping behaviors. The interaction of these parenting behaviors was not significantly related to children’s guilt. The simple effects of parental mental state language and warmth on guilt were also not significant (each predictor tested at the mean of the other because they were both mean centered). As hypothesized, the interaction between parental mental state language and warmth was significantly associated with children’s helping behaviors. As shown in Table 1, more frequent parental mental-state language was related to more children’s helping at high levels of parental warmth (i.e., 1 unit above the mean warmth), but it was related to less children’s helping at low levels of warmth (i.e., 1 unit below the mean warmth; see also Fig. 2).

We hypothesized that parental high mental state language use and low warmth would be related to children’s shame-like avoidance and decreased helping. However, these associations were not significant either for verbal or nonverbal shame-like avoidance. Although we only hypothesized warmth as moderating the effect of parental mental state language on children’s helping (see previous paragraph and Table 1), we note that the simple effect of parental warmth (at average parental mental state language) was related to decreased helping of children. This simple effect is negative for most of the range of parental mental state language, but becomes negligibly small (even negligibly positive) for parents one *SD* above the average levels of parental mental state language, *b* = 0.12, *p* = 0.78, 95% CI [− 0.71, 0.94], b^*^ = 0.04.

Finally, we hypothesized that children’s guilt would be related to increased helping behaviors and children’s shame-like avoidance would be related to decreased helping behaviors. However, guilt was not significantly related to helping behaviors. As hypothesized, verbal shame-like avoidance was significantly related to decreased helping behaviors. By contrast, children’s nonverbal shame-like avoidance was not significantly related to helping behaviors. Of note, in this model we controlled for the effects of age on children’s guilt, shame-like avoidance, and helping behaviors meaning that these effects were found after age explained some variance in children’s guilt, shame-like avoidance, and helping.

#### Testing the hypothesized model for two age groups

To test whether the expected effects were consistent across younger and older children, we ran multigroup analyses in which all the effects were first constrained across age groups. This model fitted the data significantly worse than the unconstrained model, Δχ^2^(13) = 30.48, *p* = 0.004, so we rejected the H_0_ that all the effects in the model were equal across the two age groups. Consequently, we tested which specific effects were moderated by age-group, using score tests (modification indices), each of which follows a χ^2^(1) under the H_0_ when data are normal. Because the score tests provided by lavaan (as of version 0.6–15) are not robust to nonnormality and because the “scaling factor” for each model indicated the robust test statistics should be larger than the standard test statistics, we allowed for a more liberal alpha = 0.10 to identify effects as potentially differing between two age groups, using a robust LRT and robust Wald *z* tests for follow-up confirmation.

We found evidence against equality across the two age groups for two slopes: the effect of parental warmth on helping, χ^2^(1) = 3.747, *p* = 0.053, and the effect of children’s nonverbal shame-like avoidance on helping, χ^2^(1) = 8.635, *p* = 0.003 (all other *p*s > 0.15; see Table S6 in Supplemental Materials). Thus, we fitted a final model in which we constrained all but the two effects that were flagged by modification indices, then compared it to the unconstrained model with a robust LRT. There was no evidence that this partially constrained model fit significantly worse than the unconstrained model, Δχ^2^(11) = 16.23, *p* = 0.133, so we interpreted this model that balanced parsimony and good fit. This final model fitted data well, χ^2^(15) = 18.84, *p* = 0.22, CFI = 1.00, RMSEA = 0.00 [0.00, 0.19]. All unstandardized point, *SE*, and interval estimates, as well as standardized slopes, of this model are displayed in Table 2. The model with significant and non-significant paths for two age groups is depicted in Fig. 1b.

**Table 2:** Unstandardized point and interval estimates, H_0_ tests, and standardized slopes for the hypothesized partially constrained multigroup model in older and younger age groups.

	*b*	*SE*	*p*	95% CI	b*
***Effects held equal across age groups***					
Warmth on guilt	0.22	0.14	.115	− 0.05, 0.50	0.20
Warmth on verbal shame-like avoidance	− 0.20	0.19	.284	− 0.57, 0.17	− 0.14
Warmth on nonverbal shame-like avoidance	0.05	0.04	.132	− 0.02, 0.12	0.15
MSL on guilt	− 0.01	0.01	.675	− 0.03, 0.02	− 0.05
MSL on verbal shame-like avoidance	0.00	0.03	.967	− 0.06, 0.06	0.01
MSL on nonverbal shame-like avoidance	0.01	0.01	.286	− 0.01, 0.02	0.20
MSL on helping	0.02	0.03	.568	− 0.05, 0.09	0.08
Guilt on helping	**0.57**	**0.26**	**.028**	**0.06, 1.07**	**0.23**
Verbal shame-like avoidance on helping	** − 0.52**	**0.19**	**.007**	** − 0.90, − 0.14**	** − 0.27**
Warmth × MSL on helping	**0.15**	**0.05**	**.001**	**0.06, 0.24**	**0.36**
Warmth × MSL on guilt	0.04	0.03	.197	− 0.02, 0.11	0.26
***Effects moderated by age group***					
Nonverbal shame-like Avoidance on Helping					
Younger group:	** − 5.09**	**1.58**	**.001**	** − 8.18, − 2.00**	** − 0.63**
Older group:	1.20	1.41	.395	− 1.56, 3.95	− 0.15
Group difference (older − younger):	**6.29**	**2.20**	**.004**	**1.98, 10.59**	**0.78**
Warmth on helping					
Younger group:	** − 0.97**	**0.34**	**.005**	** − 1.64, − 0.30**	** − 0.35**
Older group:	− 0.16	0.30	.597	− 0.75, 0.43	− 0.06
Group difference (older − younger):	0.81	0.44	.07	− 0.06, 1.67	0.29

*MSL* mental state language, *b* unstandardized slope, *SE* standard error of the unstandardized slope, *p* two-tailed *p* value of the Wald *z* statistic (*z* = *b*/*SE*), *CI* confidence interval around the unstandardized slope, b* standardized slope (calculated by scaling each variable by its full-sample *SD*, so results are on the same s).

Significant values are in [bold].

Regarding the slopes that differed for two age groups, we found a simple effect of parental warmth on children’s helping in younger children but not in older children. However, there was no evidence that parental warmth was differently affecting children’s helping in children of two age groups, according to robust Wald *z*-test = − 0.28, *SE* = 0.16, *p* = 0.073. Nonverbal shame-like avoidance was significantly associated with decreased helping behaviors in younger children but not in older children.

## Discussion

The aim of this study was to shed light on the socialization of self-conscious emotions in young children. Specifically, we investigated whether parental mental state language use and warmth were associated with 2–5-year-old children’s guilt and shame after a mishap and their consequent prosocial behaviors. We elicited parental and children’s behaviors using validated paradigms and we measured them using reliable (micro-)coding of lab observations. As hypothesized, we found that the combination of the frequent parental mental state language and high parental warmth was related to children’s increased helping. Children’s helping was also predicted by children’s higher guilt and lower shame-like avoidance. Most of these associations were found across toddlerhood and early childhood. These findings suggest that children’s emotional and behavioral reactions after breaking social rules and norms may be shaped by parental practices early in child development.

What psychological mechanisms explain our findings? We observed that children who helped the previously harmed experimenter more quickly had parents who frequently engaged in mentalizing and showed warmth and affection. It may be that when parents frequently talk about their own and others’ mental states, they assist children in understanding themselves and others, and in taking others’ perspective^35–37^ as well as internalizing social norms and rules^23,24^. When parents are, in addition, affectionate toward their children, they build children’s sense of self-worth^27–29^ and empathy^44^. As a result, after a mishap, children of these parents may more readily comprehend that they violated a social norm and caused harm to another person, leading them to try to repair the relationship by helping the harmed person. It is important to note that when the frequency of parental mental state language was low or average, parental warmth was negatively related to children’s helping. This suggests that only when frequent use of mental state language is combined with parental affection and support toward the child, can there be positive effects on children’s prosociality. Our finding was also in line with previous studies showing that parental mentalizing as well as parental warmth are important for children’s developing prosociality^40–43^. Our study extends this finding by showing that the combination of parental mental state language and warmth may be especially important and that and this is also the case in situations in which children broke social norms and experienced self-conscious emotions.

Our findings also shed new light on the effects of self-conscious emotions on young children’s helping behaviors. Children’s guilt was positively associated with prosocial behaviors whereas children’s verbal shame-like avoidance was negatively associated with prosocial behaviors. Nonverbal shame-like avoidance was negatively related to prosocial behaviors in toddlers, but not in children aged 4–5 years. Although we did not have a priori hypotheses about the developmental timing of these effects, this finding may be in line with empirical evidence that suggests a change in motivation for helping from toddlerhood to early childhood^45–47^. It is assumed that toddlers help due to their concern for others’ welfare^46,47^. They are not motivated by self-interest but genuinely want others to be helped^46,47^. However, around the age of four or five, children start having other motives to help as well^47^. For example, they start caring about their reputation—they prefer helping themselves rather than having others help, especially if they are being watched by others^43,47^. They also tend to help more when people can reciprocate, or when they are in-group members^48^, suggesting that external factors, such as managing reputation and personal benefit may influence older children’s helping behaviors. Taken together, this suggests that self-conscious emotions, serving as internal motivators of behaviors, may play a prominent role in prosocial behaviors, perhaps more so in toddlerhood than in childhood.

Of note, the distinction we make between guilt and shame is largely based on studies in Western countries. This distinction may apply less in non-Western collectivistic cultures^49^. In fact, there is evidence that guilt and shame are seen as very similar in collectivistic cultures [e.g.,^50,51^]. This is likely because self is construed in terms of—rather than distinct from—relationships with others (i.e., interdependent self^52^,) and it is contextually and situationally dependent. Thus, the self is not experienced as stable as in Western cultures. Experiencing the self as bad in certain contexts is then more accepted and even valued in non-Western cultures as it is seen as a motivator of self-improvement^49,53^. Cross-cultural studies suggest that, in collectivistic cultures, shame, similarly to guilt, motivates reparative actions that help rebuild relationships [e.g.,^54,55^]. Likewise, parenting practices that socialize guilt and shame may differ in different cultures. For example, Western parents express warmth through verbal and physical affection, which may cultivate self-worth^27^. However, the need for self-regard may not be universal^32^ and, thus, parental warmth may not be related to children’s shame in all cultures. Non-Western parents may put more emphasize on guidance and control, rather than warmth, in socializing their children’s emotional and behavioral reactions^56,57^. In addition, parental shaming that appears in the context of teasing, love, and intimacy may be used to socialize self-conscious emotions in non-Western cultures^58^.

More broadly, our study adds to the theory and the current discussion on biologically based versus environmentally influenced children’s emotional and behavioral reactions in social situations^59,60^. Although these emotional and behavioral reactions are likely biologically based, the findings of the current study show that these behaviors are also related to social experiences suggesting that they might be malleable. Whether social experiences or biological predispositions are foundational for the development of children’s emotional and behavioral reactions in social situations remains to be investigated. Recent evidence from twin studies shows that self-conscious emotions, such as shame, are influenced by both biological and environmental factors^61^. In addition, our study shows that the effects of social experiences on children’s self-conscious emotions and helping exist across toddlerhood and early childhood. Thus, parents seem to have an important role in socialization of children’s self-conscious emotions throughout their early development.

Surprisingly, we did not find that the combination of parental mental state language and warmth is associated with guilt, which would be in line with theory and past research on children’s guilt^23,38^. We also did not find that parental mental state language or warmth were related to shame-like avoidance. Although theory and past research suggest that children of rejecting parents who rarely show affection and support may feel unworthy and unlovable^15^, and may more easily experience shame after breaking a social norm or rule^34^, this was not the case in our sample.

Our study has several strengths, such as its precise developmental focus, its micro-coding of children’s self-conscious emotions and parental behaviors, and its investigation of two important self-conscious emotions with different social outcomes and functions. Our study also has some limitations. First, our study was correlational and the direction of the effects could not be determined. Future studies should follow children experimentally or longitudinally, so as to investigate how parental behaviors influence children’s reactions in mishap and transgression situations causally or over time. Longitudinal studies would also allow for the investigation of bidirectional effects and test whether children’s reactions also shape parental behaviors. Experiments in which parental behaviors are manipulated are needed to establish causality. Second, our analyses regarding the effects of age were exploratory, so we call for preregistered replications.

Third, we had substantial missing data for children’s guilt and shame. Although we used FIML to utilize all available information to estimate our parameters, our power to detect effects is unfortunately attenuated relative to *N* = 98 rows of complete data; however, we still expect our power to exceed 80% (see the Method for details and justification). Future studies could recruit larger samples accounting for a possibility of up to 30% missing data in the Broken toy task. Furthermore, it is plausible that for children of parents who refused to participate in the Broken Toy task or children who did not play with Teddy guilt and shame were differentially related to parental behaviors and helping, which would mean our data were missing *not* at random (MNAR; violating the assumption under which FIML provides unbiased estimates). Future research could illuminate the degree to which this may be the case, by posing alternative tasks to elicit the same guilt and shame responses. By making the reasons for missingness (e.g., parental consent, (not) playing with Teddy) more or less likely, one could estimate the degree to which such a manipulated effect moderates the relationships we estimated in this paper. The degree of bias in our results would be proportional to however large the interaction term would be; given our significant estimated interactions were mostly small-to-moderate in size, we hope that any MNAR effects introduced only a small-to-moderate amounts of bias in our results.

Fourth, although we evoked and precisely coded real parenting behaviors and children’s behaviors reflecting the experiences of self-conscious emotions, we did this in the lab. Thus, it remains to be investigated whether the same relations would be found outside of the lab, in daily life. Finally, our sample consisted mainly of parents with high socioeconomic status in a Western country. Some findings suggest that self-conscious emotions of children depend on the family’s socio-economic situation^62^ and that parental behaviors, such as mentalizing and warmth have different effects on children in families with low vs. high socioeconomic backgrounds^63,64^. Therefore, we might expect different effects in these populations. Cross-cultural evidence suggests that shame and guilt are more similar in collectivistic non-Western compared to Western cultures and that, similarly, they may involve different cognitions and behaviors in different cultures^49^. Also, there is evidence that different parenting practices are used to socialize children’s guilt and shame in Western and non-Western cultures^27,57,58^. Therefore, our study findings cannot be generalized to different non-Western cultural settings. It would be, thus, crucial to investigate the role of different parenting behaviors in socialization of guilt and shame in non-Western countries.

In summary, our study clarifies the processes by which parents might socialize children’s self-conscious emotions and their consequent behaviors after breaking social rules. The combination of parental mentalizing and warmth appears to be important for how children handle mishaps. Children of parents who frequently engage in mentalizing and who are warm and affectionate display prosocial behaviors after mishaps. Importantly, children’s own self-conscious emotions after mishaps may also be influential in how prosocial children behave toward others. These findings pave the way for future research on the interplay between social experiences and individual differences in children’s emotional and behavioral responses following the violation of social norms and rules.

## Methods

### Participants

Participants were 98 children (52% girls) aged 2–5 years (*M* = 48.66 months, *SD* = 13.50 months) who were accompanied by one parent (82.60% mothers) aged 22–48 years (*M* = 36 years, *SD* = 6 years) to the Family laboratory of the University of Amsterdam. We used the R package pwr^65^ to estimate the minimum sample size (*N* = 60) needed to obtain 80% power to detect a medium interaction effect (Cohen’s *f*^2^ = 0.15), the calculate of which assumes we would use multiple regression. Because we used structural equation modelling (SEM) to conduct a path analysis, we performed a subsequent power analysis that was also more informed by our estimated parameters. Using the standardized estimates of simple effects (i.e., omitting estimated interaction effects) as population parameters, we additionally specified the hypothesized interaction effects as 0.3 for helping and − 0.3 for guilt. Using^66^ method implemented in^67^ web app for SEM power analysis, a minimum *N* = 75 to detect the interaction effect on helping and a minimum *N* = 66 to detect the interaction effect on guilt. If we had *N* = 98 complete observations, we would have had 89% and 93% power to detect these two hypothesized medium interaction effects. However, our actual power was diminished both by incomplete data and by calculating “sandwich” standard errors that are robust to nonnormality. Although it is impossible to know exactly how much power was diminished without knowing the true missing mechanism, the true distribution of the variables (’ residuals), or all other true population parameters in our model—we are nonetheless confident that we had sufficient power to detect our hypothesized effects because we observed at least *n* = 66 out of *N* = 98 values for each variable in our model, which was the minimum complete-data *N* we found above for 80% power. Thus, the additional 98 − 66 = 32 parent–child dyads with incomplete data can be expected to increase our power above 80%.

**Figure 3:**
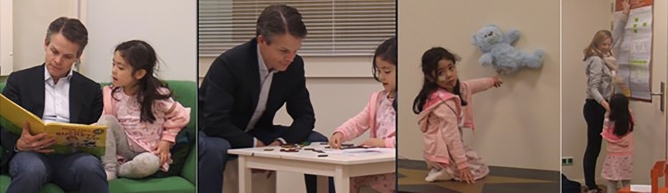
Setting of the tasks: (**a**) Picture-book reading task. (**b**) Puzzle task. (**c**) Broken toy mishap task. (**d**) Helping task.

Families were recruited through public elementary schools, social media, and public places for children (museums, zoo) for a larger study about self-conscious emotions in children. Parents received information letters about the study and those who left their contact information or contacted the research team were invited to the lab. Parents were 82% Dutch, relatively highly educated: 38% graduated from a university, 38% graduated from college, and 24% finished a high school or vocational education. Most of the parents were working, full-time (30%) or part-time (59%). The household income of the parents included in the study was representative of Dutch population with an average household income per month being equal to the average household income of Dutch families in general (CPB Netherlands Bureau for Economic Policy Analysis), *Median* = 3 (4000–6000 euros), on the scale from 1 = less than 1000 euros to 7 = more than10000 euros. Parents signed parental informed consent for their children and themselves prior to the participation in the study. The study was approved by the Ethics Review Board of the Faculty of Social and Behavioral Sciences of the University of Amsterdam. The study was performed in accordance with the guidelines and regulations of the Declaration of Helsinki on human experimentation. Informed consent was obtained to publish images in Fig. 3 in an online open access publication.

### Procedure and measures

Children visited the research lab with one parent. We had four wall-mounted high-quality cameras on each wall of the room with which we recorded the whole procedure. During the lab visit, among other tasks, a picture book reading task was conducted to elicit parental mental state language, a puzzle task was used to measure parental warmth, a broken toy mishap task was used to evoke children’s self-conscious emotions after transgression, and helping tasks were conducted to elicit children’s helping behaviors (Fig. 3).

#### Spontaneous parental mental state language

During the picture book-reading task, the parent was asked to sit on the sofa with their child and read a picture book as they would do at home^68^. The words in the book were stricken through so that only pictures were visible in order to encourage parents’ spontaneous talk. An age-appropriate book How Full Is Your Bucket? For Kids^69^ was selected for reading because it included multiple scenes that depicted social events and emotional content that parents could talk about thereby eliciting variations in parents’ mental state language usage. Parents’ language during the book reading was transcribed verbatim from the video recordings and transcripts were then coded using a validated coding system for parental mental state language usage^70^. Three categories of mental state language were coded: (1) emotions (e.g., happy, angry, worried), (2) cognitions—knowledge and beliefs (e.g., think, know, imagine), and (3) intentions/wishes (e.g., want, wish, like). The expressions such as “I don’t know” were not coded as mental state language comments, because they typically mean: “I can’t answer”. Also, utterances that included mental state language but were repeated more than once by the parent were not coded as mental state language after the first mention. Comments in which parents repeated what the child said were also not coded as mental state language. Mental state language was coded for any utterance involving mental state terms, no matter if these terms referred to the characters in the book, the parent, the child, or anyone else to account for any mental state language use during the book-reading. The number of mental state language utterances of all three categories (i.e., emotions, cognitions, and intentions/wishes) were summed up in a total number of mental state language utterances. To control for parental verbosity, transcripts were also coded for the total number of utterances so the number of mental state language utterances could be converted to proportions (total mental state language utterances/total utterances). Mental state language was coded by two master students who were extensively trained in the coding system. Inter-rater reliability was established on the proportion of total mental state language utterances from 20 (24%) transcripts. Reliability was excellent, two-way random absolute agreement ICC = 1.00.

#### Parental warmth in situations of children’s success and failure

Parental warmth was coded during the success and failure puzzle task^71^, which was adapted for the present study. The task consisted of five puzzles: three success and two failure trials that were given alternately in a standardized order. In all sets, the child needed to match coloured puzzle pieces to the picture on a worksheet. The success puzzle trials had fewer pieces to be matched than the unsolvable puzzle trials. In addition, for the unsolvable puzzle trials, one piece of the puzzle was removed to make sure that the puzzle could not be solved. Before starting each trial, the experimenter told children that they have 2 min to complete the puzzle. The experimenter showed a timer to the child and said that she would do the timing and demonstrated to the child how the timer would ring after 2 min. The experimenter also explained to the child that the child would succeed if they complete the puzzle before the timer rings and that they would fail on the task if they would not complete the task by the time the timer rings. To manipulate the success and failure, the experimenter controlled the speed of the timer, letting the child finish before the timer rung in the success trials and ringing the timer after the child was almost done with the unsolvable puzzle (when there were 2–3 matches left to be made). The task started with an instruction puzzle trial in which the experimenter explained the task to the child and made sure the child understood the instructions. All the trials started with the success puzzle. To ensure that the task was finished on a positive note, a third success puzzle was added but not included in the coding. For the success trials, the experimenter said “Time is up. You succeeded in completing the puzzle before the bell.” For the failure trials, the experimenter said: “Time is up. You failed to complete the puzzle before the bell”. After giving feedback about success or failure, the experimenter remained silent for 30 s to allow the parent to react to the puzzle outcome. After the task was done, the experimenter debriefed the child by saying that she just noticed that some of the pieces of the puzzle were missing and that this is why the child could not solve the failure puzzle trials.

During the procedure, the child was seated on a child-sized chair and table and the parent was seated next to the child. At the beginning of the task, parents received the instruction that they could help their child with the puzzle but should not touch the puzzle. They were also instructed to react to the outcome of each trial after the timer rang as they would usually do when the child works on a task and fails or succeeds. Warmth was coded during the first success and first failure puzzle because we observed most of the variations in parental and children’ behaviors in the first two trials. We used the validated coding system Meso Behavioral Rating System for Families (MeBRF)^72^ to code parental warmth. The puzzle task was divided in time intervals of 1 min (the second minute of the puzzle task and the reaction lasted less than 1 min and the coding was adjusted to the duration). Parental warmth was coded on a 5-point Likert scale, 1 = low frequency/intensity of parental warmth, 5 = high frequency/intensity of parental warmth. The rating for each minute reflected the summary evaluation of parental warmth for that minute. Warmth was operationalized as the frequency and the intensity with which the parent proactively communicated affection and support toward the child. Two aspects of parental warmth were coded: verbal and non-verbal warmth. Verbal warmth included praise (e.g., you did great), spontaneous and enthusiastic comments about the child or their performance (e.g., yes, that is the way to go), supportive comments (e.g., this looks good, go on). Nonverbal warmth included physical affection (e.g., a cuddle, a hug), smiling toward the child, and supportive gestures (e.g., high-fives, thumb up). The coding system is displayed in Table S1.

Three master students were extensively trained into this coding system. Eleven observations were coded by all students (12% triple-coded). The scores were averaged across intervals and across verbal and non-verbal warmth into the composites reflecting warmth during the success and failure puzzle trials. Reliability was good to excellent, two-way random absolute agreement ICC = 0.74 for warmth during the failure trial and two-way random absolute agreement ICC = 0.85 for warmth during the success trial. Warmth during the success and failure trial was highly correlated, *r*(*n* = 84) = 0.59, *p* =  < 0.001 and was further averaged into total parental warmth.

#### Children’s expressions of self-conscious emotions after transgression

Children’s expressions of self-conscious emotions were coded during the Broken Toy mishap task^1,9,73^. Before the start of the task, parents received an instruction card that informed them about the task and the deception and the task was performed only if the parent gave an additional approval. Four parents did not give their approval. Parents were instructed to remain seated at the desk on the other side of the room and fill in questionnaires on the lap top. They were also instructed to remain as neutral as possible if the child tried to involve the parent during the task. For the task, the experimenter brought in a teddy bear called Teddy and told the child that Teddy was her favorite toy from the childhood emphasizing the emotional value of the toy. The experimenter then attached Teddy to the wall with a Velcro patch and told the child that they can play with Teddy but that they need to be careful with it while the experimenter is gone “ to pick up some papers”. Teddy was rigged so that his arm and leg would fall off when the child would try to pull Teddy from the wall to start playing with it. The child did not know that Teddy was rigged, thus, they were made to think that they broke Teddy. Around 2 min after the child pulled and “broke” Teddy, the experimenter returned to the room and gave five cues with 15 s intervals in between in a standardized order to encourage the child to respond to the situation. First, the experimenter said loudly in front of the room before entering “Alright, thanks a lot and see you soon!” so the child knew the experimenter was coming back. When the experimenter entered the room, she looked at the broken Teddy with a neutral facial expression. Next, she asked “What happened to Teddy?”. After 15 s, she again asked: “Why did his arm/leg fell of?”. Finally, she said: “Teddy was my favorite bear.” After these five cues, the experimenter debriefed the child by saying: “Oops, I forgot that Teddy was already broken! I can fix him; I will be right back with a repaired Teddy.” After leaving the room, the experimenter returned with an identical, non-broken Teddy and said: “See, he is as good as new. Now Teddy is happy again, I am happy again and you can be happy again as well.”. If the child showed severe signs of distress (e.g., looking like they would start crying) at any point during the task, the experimenter skipped any left cues and immediately debriefed the child for ethical reasons.

We coded children’s expressions of self-conscious emotions using a validated coding system^1,9^. We also added verbal codings reflecting self-conscious emotions based on^74^. We micro-coded videos second-by-second (in slow motion) for following behaviors: gaze, head, and body aversion, positive vs. non-positive facial expressions (captured by lip corners going up or not and indicating a smile or no smile), repairing of Teddy, Talking about Teddy, and verbal expressions related to the situation (e.g., taking responsibility for breaking teddy, comforting experimenter). All nonverbal behaviors were coded as state events continuously throughout the observation and duration of these behaviors was calculated and then transformed into percentage to account for the whole duration of the observations (which differed between children). Verbal behaviors were coded every time they occurred and total number of verbal behaviors was calculated. More detailed descriptions and examples of coded behaviors are displayed in Table S2. Similarly to^9^, we performed a principal component analysis with Oblimin rotation to find out which coding behaviors load onto the same factor, reflecting a specific self-conscious emotion. The results of the principal component analyses are reported in Table S3. In line with the theory and past research, we found that the coded behaviors were organized in three patterns reflecting two self-conscious emotions: guilt and shame. For shame, unlike in previous studies, we found a distinction between verbally and non-verbally expressed shame. These three components explained 64% of variance between children. The behaviors reflecting each of these three emotional patterns were standardized and averaged into composite scores to operationalize guilt, verbal and non-verbal shame-like avoidance. Guilt was a composite of repairing Teddy, expressing concern for the experimenter, and comforting the experimenter. Verbal shame-like avoidance consisted of high latency to talk to the parent and/or the experimenter (i.e., number of seconds until the child started talking), and fewer verbal expressions that indicate taking responsibility or adopting the experimenter’s perspective. Non-verbal shame-like avoidance consisted of gaze, head and body aversions (looking away and turning the head or body away) during non-positive facial expressions from the parent/experimenter. The behaviors were coded by two master students who received an extensive training in coding self-conscious emotions in children by the first author. Their inter-rater reliability was established on 12 observations (12 double-coded pairs of observations). In addition, two master students were trained to code for reliability purposes by the first author and coded 10 observations; inter-rater reliability was established between two of them and between each of them and the original coders resulting in 26 double-coded pairs. In total, we, thus, calculated inter-rater reliability, which was acceptable to excellent, on 38 double-coded pairs of observations. Cohen’s kappa corrected for kappa max was κ = 0.61 for gaze aversion during neutral, negative, and positive facial expressions, κ = 0.74 for head aversion during neutral, negative, and positive facial expressions, κ = 0.83 for body aversion during neutral, negative, and positive facial expressions, κ = 0.95 for not talking about Teddy, κ = 0.83 for talking about Teddy, κ = 0.95 for repairing Teddy.

#### Children’s helping behaviour

Instrumental and empathetic helping tasks^1^ were used to measure children’s prosocial helping. In the instrumental helping task**,** the experimenter took a poster and tape and said that she would hang the poster on the lab door. The child was seated near the door so they could see what the experimenter was doing. While the experimenter was hanging the poster, she “accidentally” dropped the tape on the floor. After the tape fell on the floor, the experimenter gave six cues to prompt help from the child: (1) right after dropping the tape, the experimenter said: “Ooops!”; (2) the experimenter said: “Oh my tape, it fell on the floor” but did not look at the child; (3) the experimenter held the poster with one hand and turned to the child and said: “My tape, I need it back!”; (4) the experimenter leaned down in an attempt to reach for the tape while still holding the poster with one hand and said: “Oh, my tape.”; (5) the experimenter tried to reach for the tape again and said: “[child’s name], can you help me?”; and (6) the experimenter again tried to reach for the tape and said: “[child’s name], can you help me get my tape back, please?”. The experimenter waited for approximately 6–7 s after each cue in order to allow the child to help. Once the child helped, the experimenter stopped with the cues, hung the poster on the wall, and finished the task. If the child failed to respond to all cues, the experimenter picked up the tape and hung the poster.

In the empathetic helping task, the goal is for the child to bring a blanket to the experimenter when she shows signs of being cold. Before the start of the task, in between other tasks of the lab visit, the experimenter introduced a blanket to make sure every child understood that the experimenter could get warm by covering herself with the blanket when she was cold. In this demonstration, the experimenter shivered and said: “Brrrr, I am cold. I will take a blanket to cover for a bit because it makes me warm.” The experimenter then took the blanket, covered herself for a minute and afterwards left the blanket on the table near the child’s reach. The empathetic helping task started by the experimenter sitting on the sofa and telling the child that she needs to write something down on her papers. The experimenter then showed six cues while sitting and writing: (1) the experimenter loudly shivered and said “Brrr” without looking at the child; (2) the experimenter said: “I am cold” without looking at the child; (3) the experimenter looked at the child and said: “I need something to help me feel warm”; (4) the experimenter leaned toward the blanket on the table and said: “Oh blanket!”; (5) the experimenter leaned toward the blanket and then looked at the child saying: ““[name of the child], please will you help?”; (6) the experimenter again leaned towards the child and said: “[name of child], please will you help me with my blanket?”. As in the instrumental helping task, the experimenter waited for approximately 6–7 s after each cue in order to allow child to react. Once the child helped, the experimenter stopped with the cues, covered with the blanket, and finished the task. If the child failed to respond to all cues, the experimenter picked up the blanket and covered herself on the sofa.

Children’s helping behavior was coded so that children who helped after the first cue received the highest score of 6 reflecting the highest level of prosocial behaviors. The scores gradually decreased with each new cue so that the child received the lowest score of 0 if they did not help at all. Two master students were trained to code helping behaviors. Twenty observations (23%) were double-coded to establish inter-rater reliability. Reliability was excellent, two-way random absolute agreement ICC = 1.00 for the instrumental helping task and two-way random absolute agreement ICC = 0.99 for the empathetic helping task. The scores on the instrumental and empathetic task were strongly correlated, *r*(*n* = 77) = 0.46, *p* =  < 0.001, and averaged into the score for total helping behaviors.

### Data analyses

Data were checked for univariate outliers (± 3 *SD*) and outliers were Winsorized modifying their values to the closest observed values in the range of ± 3 *SD*^60^. Two outliers for the guilt expressions and one outlier for parental mental state language above the range of ± 3 *SD* were Winsorized. There were no multivariate outliers according to Mahalanobis or Cook’s distances. Data were also checked for normality using histograms and skewness and kurtosis values. All variables except children’s expressions of guilt and helping were normally distributed. Because we observed deviations from normality, we used robust maximum likelihood (ML) estimation (i.e., maximum likelihood with robust standard errors, MLR) in our path models in order to more accurately estimate standard errors^75^. Pearson’s correlations were calculated to examine the zero-order linear associations between the study variables.

Missing values appeared for several reasons. In the case of the puzzle task, the task was not coded due to: bad video quality (n = 1); the task was not performed due to a procedural error (n = 3); the child not wanting or not being able to engage with the puzzle task (n = 4). In the case of the picture-book reading task, the task was: not recorded due to a procedural error (n = 1); not coded when there was no sound due to a procedural error (n = 4); due to a foreign language (other than Dutch) the mother spoke to the child (n = 5); not transcribed due to bad sound quality (n = 3) or video quality (n = 1); not performed due to a procedural error (n = 1); or the child did not want to engage in book reading (n = 1). For the Broken toy task, we had missings due to the parent not agreeing to perform this task (n = 4); the child did not play with and break Teddy (n = 14); not performing the task during the lab visit due to parent/child wanting to finish the visit (n = 7); Teddy breaking before the experimenter left the room (n = 2); Teddy not breaking while playing (n = 3); or the child repairing Teddy before the experimenter returned to the room (n = 2). In the case of helping tasks, the tasks were not coded: when there was no sound due to a procedural error (n = 2); when it was not performed because the parent/child wanted to end the visit (n = 9); or the parent intervened in both tasks (n = 1). In some cases, we had missings on one of the prosocial tasks but not in the other prosocial task. In this case, we used the score from the task that was performed. In total, there were eight missing values for warmth, 16 missing values for mental state language, 32 missing values for self-conscious emotions, and 12 missing values for helping behaviors. Importantly, in all the models, we used full information maximum likelihood (FIML) to estimate parameters using all available information from incomplete data; thus, the whole sample (*N* = 98) was used to estimate the effects of interest. FIML estimation assumes data are missing at random^76^, but in practice it is reasonable to expect that data go missing for a mixture of (completely) random and nonrandom reasons^77^. The MAR assumption is difficult to verify in a cross-sectional study. We checked whether children who had missing values on each of the constructs differed on all other study constructs from children who had data on that construct. We found that children with missing or observed self-conscious emotions did not significantly differ on all other study constructs. We found this pattern of results for all study constructs (all *p*s > 0.087), except in the case of missings on parental mental state language. Children who had missing data on parental mental state language showed more verbal shame-like avoidance (but not other self-conscious emotions) than children for whom we had data on parental mental state language (*p* = 0.010). Given this difference, our findings suggest that our data may be missing at random conditional on this modeled variable^76^.

To test our hypotheses that parenting behaviors are associated with children’s self-conscious emotions and prosocial behaviors and that children’s self-conscious emotions are related to their prosocial behaviors, we fitted a path model within the SEM framework. Specifically, we tested our hypothesises that (1) the interaction between parental mental state language and parental warmth is associated with children’s guilt; (2) parental warmth and parental mental state language is associated with children’s verbal and nonverbal shame-like avodiance; (3) the interaction between parental mental state language and parental warmth is associated with children’s helping; and (4) children’s guilt and verbal and nonverbal shame-like avoidance are related to their helping behaviors. Furthermore, to explore possible age differences in these associations, we fitted a multigroup path model with which we tested whether the associations of interest were equal or not across two age groups: for younger children aged 2–3 years and for older children aged 4–5 years.

The evaluation of the models was carried out in R using package lavaan^78^. To obtain parameter estimates and evaluate the goodness of fit of the path model, we used the robust maximum likelihood method. The maximum likelihood provided a χ^2^ test of model fit. A significant χ^2^ value indicates a discrepancy between the model-implied and the observed covariance matrices that is greater than sampling error would account for in most samples (i.e., 95% confidence level), indicating that the model does not fit the data well. In addition to χ^2^, we used the root mean square error of approximation (RMSEA)^79^, and the comparative fit index (CFI)^80^. RMSEA less than 0.01, 0.05, and 0.08 indicates excellent, good, and approximate fit, respectively. CFI values above 0.95 indicate good fit^81^. We compared nested models by means of robust (log-)likelihood ratio tests (LRT) at the significance level of α = 0.05, where a significant χ^2^ test indicates the more constrained model fits the data significantly worse.

First, we fitted the hypothesized model in which we constrained the non-hypothesized paths to zero (i.e., the interaction between parental mental state language and warmth on verbal and nonverbal shame) and investigated whether this resulted in a significant decrease in model fit compared to the saturated model. If this decrease was not significant according to the robust LRT, this would imply that the more parsimonious model could be retained. This procedure resulted in a model in which only the hypothesized paths were left.

Next, we fitted the multi-group model to test whether the modeled associations differed significantly for younger (2–3 years old, *n* = 50) and older (4–5 years old, *n* = 48) children. We tested whether the effects in the model are equal across the age groups by imposing cross-group equality constraints on the estimates of each path coefficient in the model^82^. We used a robust LRT^83^ to obtain a single omnibus test of the H_0_ that paths do not depend on (i.e., are not moderated by) age group. A non-significant test indicates that the constrained model does not worsen the fit to the data and, thus, failure to reject the H_0_ of no moderation by age. A significant test indicates that the magnitude or the direction of (at least some of) the effects differ between the age groups. When this was the case, we used score tests (modification indices, χ^2^ statistics which are asymptotically equivalent to LRT^84^; to identify which of the slopes should not be constrained to equality. Again, if the LRT was insignificant, it meant we could not reject the H_0_ that the constrained effect did not differ across age groups. Finally, we compared the unconstrained model to the model in which we constrained only the paths for which we did not reject H_0_, representing partial moderation by age.

### Supplementary Information

Supplementary Tables.

## Data Availability

All data and code are available on Open Science Framework: https://osf.io/hdypw/?view_only=e9f10ad6a75349d085c88ae12055ce36. All protocols used in our procedure are available on Open Science Framework: https://osf.io/zkdm6/?view_only=9ccda725e59f41b9bca1225d8c4b6e90.
