# Author Correction: Parental socialization of guilt and shame in early childhood

**DOI:** 10.1038/s41598-023-42125-x

**Published:** 2023-09-07

**Authors:** Milica Nikolić, Eddie Brummelman, Bram Orobio de Castro, Terrence D. Jorgensen, Cristina Colonnesi

**Affiliations:** Research Institute of Child Development and Education, University of Amsterdam, Amsterdam, The Netherlands

Correction to: *Scientific Reports* 10.1038/s41598-023-38502-1, published online 20 July 2023

The original version of this Article contained an error in the sentence, in the Methods section, under the subheading ‘Participants’,

“Participants were 98 children (52% girls) aged 2–5 years (*M* = 48.66 months, *SD* = 13.50 months) who were accompanied by one parent (82.60% mothers) aged 22–48 years (*M* = 36 years, *SD* = 6 years) to the [blinded for review] laboratory of the University of [blinded for review].”

now reads:

“Participants were 98 children (52% girls) aged 2–5 years (*M* = 48.66 months, *SD* = 13.50 months) who were accompanied by one parent (82.60% mothers) aged 22–48 years (*M* = 36 years, *SD* = 6 years) to the Family laboratory of the University of Amsterdam.”

The original Article has been corrected.

