# Supplementary material for: Parental socialization of guilt and shame in early childhood
Source: Sci Rep. 2023 Jul 20;13:11767. doi: 10.1038/s41598-023-38502-1 (PMC10359293; doi:10.1038/s41598-023-38502-1)
Supplement: Supplementary file 1 — Supplementary Tables. [file 41598_2023_38502_MOESM1_ESM.docx]

**Supplemental Material**

Table S1

*Warmth Coding Scale*

| Score | Description | Examples |
| --- | --- | --- |
| 1 | No traces of affection or support initiated by the parent. | Parent is talking to the child with a neutral tone, parent may smile, but only as a reaction to the child smiling (not initiated by the parent). |
| 2 | Low frequency and low intensity affective or supportive behaviors initiated by the parent. | Parent shows one or two slight smiles or one smile and one supportive comments: yes, go on |
| 3 | A few (3-4 mild affective and supportive behaviors or 2 high-intensity (clear and sincere) affective and supportive behaviors initiated by the parent. | Parent smiles and nods a few times and says “yes, go on” or parent gives a compliment such as “good job, that was fast!”, and a high five or a big smile. |
| 4 | Many fairly intense affective and supportive behaviors (4 behaviors of high intensity or more than 4 behaviors out of which two behaviors were of high intensity). initiated by the parent. | Parent gives compliments with enthusiasm, e.g., “wow, you are so good at this!”, laughing together, physical affection such as a hugs and kisses. |
| 5 | Many (more than four) high-intensity affective or supportive behaviors initiated by the parent. | Parent often gives warm, sincere and enthusiastic compliments, praise or encouragement and big sincere smiles, laughs together with the child, gives hugs or kisses. |

Table S2

*Self-Conscious Emotions Behavioral Coding*

| Behavior | Description | | Example |
| --- | --- | --- | --- |
| Gaze aversion | Averted gaze from E | | The child looks away (e.g., at the floor) after looking at E |
| Head aversion | Averted head from E | | The child averts or hides their head (e.g., cover face with hands) after facing E |
| Body aversion | Averted body from E | | The child averts or hides their body after facing E |
| Lips | Indicating a smile or no smile | | Lip corners up; indicating smile. Lip corners neutral or down; not indicating smile |
| Repairing | Repairing Teddy | | The child is trying to put the arm, leg, or stuffing back |
| Talking | Talking about Teddy to parent and E (once E entered the room) | | “Look, Teddy is broken” |
| Verbal expressions | Taking responsibility | | “I made the arm/leg fall of” |
|  | Acknowledging feelings/ taking perspective of E | | “She (E) is going to be sad” |
|  | Comforting E | | “I can fix it” |
|  | Concern what will happen to E or Teddy | | “How will you fix Teddy?” |
| Emotion | | Behavior | |
| Guilt | | Repairing; comforting E; concern what will happen to E or Teddy | |
| Verbal shame-like avoidance | | Latency to talk about Teddy; lack of acknowledging feelings/ taking perspective of E; lack of taking responsibility | |
| Non-verbal shame-like avoidance | | Gaze aversion; head aversion; body aversion (while not smiling) | |

Table S3

*Principal Component Analyses of Coded Behaviors in the Broken Teddy Task*

|  | Nonverbal shame-like avoidance | Guilt | Verbal shame-like avoidance |
| --- | --- | --- | --- |
| Latency to talk |  |  | .78 |
| Taking responsibility |  |  | -.66 |
| Taking perspective |  |  | -.66 |
| Comforting the experimenter |  | .87 |  |
| Expressing concern |  | .86 |  |
| Gaze aversion | .90 |  |  |
| Head aversion | .93 |  |  |
| Body aversion | .75 |  |  |
| Repairing Teddy |  | .57 |  |

*Note.* Loadings of individual items on each factor are shown when they are above .30

Table S4
*Descriptive Statistics and Correlations for Parental Warmth, Mental State Language, Children’s Self-Conscious Emotion Expressions, Prosocial Behaviors, and Age*

|  | *n* | *M(SD)* | 1 | 2 | 3 | 4 | 5 | 6 |
| --- | --- | --- | --- | --- | --- | --- | --- | --- |
| 1. Parental warmth | 90 | 2.33 (0.62) | — | — | — | — | — | — |
| 2. Parental MSL | 82 | 21.43 (6.81) | -.01*** | — | — | — | — | — |
| 3. Guilt | 66 | -0.04 (0.71) | .17* | .12 | — | — | — | — |
| 4. Verbal shame-like avoidance | 66 | 0.04 (0.89) | −-.14*** | -.02** | .07** | — | — | — |
| 5. Nonverbal shame-like avoidance | 66 | 0.52 (0.21) | .14** | .16** | -.15** | .03** | — | — |
| 6. Helping | 86 | 3.90 (1.72) | -.20* | .01 | −.23** | -.25 | -.12 | — |
| 7. Age in months | 92 | 48.66 (13.50) | -.25* | .15 | ..45** | .08 | -.12 | .28* |

*Note.* MSL = mental state language. Guilt, verbal and non-verbal shame-like avoidance are average composites of indicators which were standardized.

Table S5
*Descriptive Statistics and Correlations for Parental Warmth, Mental State Language, Children’s Self-Conscious Emotion Expressions, and Prosocial Behaviors for Younger and Older Group of Children*

|  | Children aged 2-3 years | | | | | | | Children aged 4-5 years | | | | | | |
| --- | --- | --- | --- | --- | --- | --- | --- | --- | --- | --- | --- | --- | --- | --- |
|  | *n* | *M(SD)* | 1 | 2 | 3 | 4 | 5 | *n* | *M(SD)* | 1 | 2 | 3 | 4 | 5 |
| 1. Parental warmth | 39 | 2.47 (0.65) | — | — | — | — | — | 46 | 2.20 (0.55) | — | — | — | — | — |
| 2. Parental MSL | 36 | 20.05 (5.48) | .05*** | — | — | — | — | 40 | 23.02 (7.06) | -.07 | — | — | — | — |
| 3. Guilt | 35 | -0.29 (0.62) | .29* | .29 | — | — | — | 30 | 0.26 (0.71) | .16 | -.07 | — | — | — |
| 4. Verbal SlA | 35 | 0.04 (0.91) | −-.28*** | .16** | -.06** | — | — | 30 | 0.05 (0.89) | .02 | -.20 | -.27 | — | — |
| 5. Nonverbal SlA | 35 | 0.53 (0.18) | .20** | .45*** | .44**** | .08** | — | 30 | 0.50 (0.25) | .04 | -.03 | -.15 | -.03 | — |
| 6. Helping | 37 | 3.30 (1.98) | -.24 | .17 | .26** | -.23 | -.38* | 45 | 4.27 (1.34) | -.04 | -.10 | .01 | -.31 | .30 |

*Note.* MSL = mental state language. SlA = shame-like avoidance.

Table S6

*Score Tests (Modification Indices) for Equality Constraints in Multigroup Model*

| Tested Effect | χ^2^(1) | *p* |
| --- | --- | --- |
| **Warmth on Helping** | **3.747** | ***0.053*** |
| MSL on Helping | 0.142 | 0.706 |
| Warmth × MSL on Helping | 1.125 | 0.289 |
| Guilt on Helping | 0.453 | 0.501 |
| Verbal shame-like Avoidance on Helping | 0.012 | 0.912 |
| **Nonverbal Shame-like Avoidance on Helping** | **8.635** | **0.003** |
| Warmth on Guilt | 0.339 | 0.560 |
| MSL on Guilt | 0.692 | 0.405 |
| Warmth × MSL on Guilt | 0.585 | 0.444 |
| Warmth on Verbal Shame-like Avoidance | 0.453 | 0.501 |
| MSL on Verbal Shame-like Avoidance | 0.104 | 0.747 |
| Warmth on Nonverbal Shame-like Avoidance | 0.168 | 0.682 |
| MSL on Nonverbal Shame-like Avoidance | 2.076 | 0.150 |
